# Temporal dynamics of uterine immune microenvironment remodeling in a murine model of adenomyosis

**DOI:** 10.1093/molehr/gaaf057

**Published:** 2025-11-26

**Authors:** Marlyne Squatrito, Julie Vervier, Laëtitia Bernet, Alessandra Camboni, Marie-Madeleine Dolmans, Carine Munaut

**Affiliations:** Laboratory of Biology of Tumor and Development, GIGA-Cancer, University of Liège, Liège, Belgium; Laboratory of Biology of Tumor and Development, GIGA-Cancer, University of Liège, Liège, Belgium; Department of Obstetrics and Gynecology, Hôpital de la Citadelle, University of Liège, Liège, Belgium; Laboratory of Biology of Tumor and Development, GIGA-Cancer, University of Liège, Liège, Belgium; Gynecology Research Unit, Institut de Recherche Expérimentale et Clinique, Université Catholique de Louvain, Brussels, Belgium; Anatomopathology Department, Cliniques Universitaires Saint-Luc, Brussels, Belgium; Gynecology Research Unit, Institut de Recherche Expérimentale et Clinique, Université Catholique de Louvain, Brussels, Belgium; Gynecology Department, Cliniques Universitaires Saint-Luc, Brussels, Belgium; Laboratory of Biology of Tumor and Development, GIGA-Cancer, University of Liège, Liège, Belgium

**Keywords:** adenomyosis, murine model, uterine immune microenvironment, macrophage polarization, T-cell subset reprogramming, interleukin-6/STAT3 signaling

## Abstract

Adenomyosis—the ectopic presence of endometrial glands and stroma within the myometrium—affects reproductive-age women and is associated with pain, bleeding, and subfertility, yet the immune events that precede pregnancy remain poorly defined. To address this, we investigated how the uterine immune microenvironment evolves before conception in a tamoxifen-induced murine model of adenomyosis. At 1- and 3-months post-induction, we analyzed uterine tissue by immunofluorescence, flow cytometry, and quantitative polymerase chain reaction. We found that adenomyotic uteri exhibited a sustained elevation of interleukin-6 messenger RNA, a transient interleukin-10 rise at 1 month, and stable cyclooxygenase-2 levels. Interleukin-6 receptor messenger RNA and signal transducer and activator of transcription 3 messenger RNA were both transiently downregulated at 1 month and returned to control levels by 3 months. Early in disease development, total macrophage numbers declined and displayed an alternative (M2) activation phenotype, followed by a selective loss of classically activated (M1) macrophages at later stages. B lymphocytes were consistently enriched, indicating enhanced humoral activity. Although overall T-cell counts remained stable, the CD3^+^ compartment underwent a marked shift from double-negative T lymphocytes toward T-helper and cytotoxic subsets, coinciding with the transient signaling changes. Limitations of our study include reliance on a single animal model, analysis at only two timepoints, and a lack of functional assessment of regulatory T cells. Future work should incorporate finer temporal profiling, single-cell transcriptomics, and validation in human tissues. These findings highlight dynamic innate–adaptive crosstalk as an early driver of adenomyosis pathology and suggest that targeting interleukin-6-mediated pathways may inform biomarker development and novel immunomodulatory interventions.

## Graphical Abstract

**Figure:**
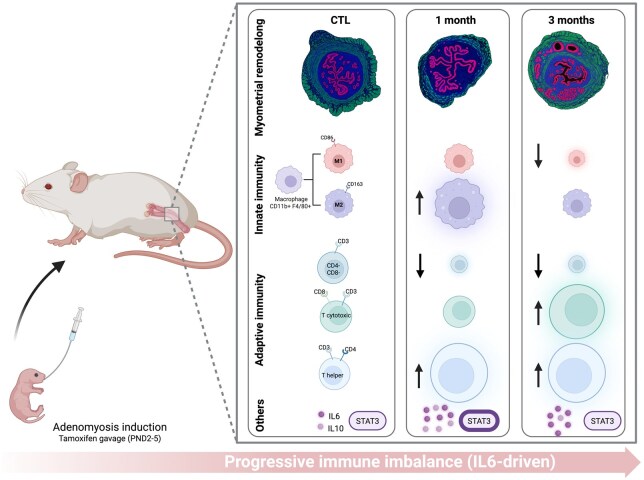
**Adenomyosis progression in mice is associated with macrophage M2 shift, altered T-cell subsets, and IL-6/STAT3–driven immune imbalance.** CTL, mice treated with vehicle; “1 month” and “3 months” refer to the time post-tamoxifen treatment. Created in BioRender. Munaut, C. (2025) https://BioRender.com/t13843g

## Introduction

Adenomyosis was first identified at the end of the 19th century in uteri examined post-hysterectomy (Bird *et al.*, 1972). More recently, advances in medical imaging have enabled non-invasive diagnosis, underscoring that adenomyosis also affects younger, reproductive-age women (Pinzauti *et al.*, 2015; Chapron *et al.*, 2017; García-Solares *et al.*, 2018). Clinically, the condition is often associated with pelvic pain, abnormal uterine bleeding, and multiparity (Vannuccini and Petraglia, 2019; Vercellini *et al.*, 2025). Since the initial theory positing that endometrial tissue penetrates the myometrium through a compromised junctional zone (Bergeron *et al.*, 2006; Stratopoulou *et al.*, 2021), numerous molecular pathways have been explored to elucidate pathogenesis.

Over the past decade, murine models, particularly tamoxifen-induced models, have emerged as key research platforms for dissecting adenomyosis mechanisms (Parrott *et al.*, 2001; Green *et al.*, 2005). Although multiple mechanisms contribute to lesion progression, immune dysregulation has emerged as a critical driver (Li *et al.*, 2024). Bourdon *et al.* highlighted immunological disturbances and aberrant Notch1 signaling near or during the implantation window (Bourdon *et al.*, 2021, 2024). However, our group was the first to demonstrate a direct fertility impairment in tamoxifen-induced adenomyosis mice, documenting disrupted estrous cyclicity, prolonged estrus, impaired folliculogenesis, and notably smaller litter sizes and numbers (Squatrito *et al.*, 2024). We further linked these reproductive defects to progesterone resistance and reduced endometrial receptivity, as evidenced by downregulation of progesterone receptor expression, HoxA10, and integrin β3.

Despite these advances, the precise pathways by which adenomyosis impairs fertility remain incompletely defined. Several studies have implicated eutopic-endometrial immune dysregulation as a key contributor (Barrier *et al.*, 2004; Campo *et al.*, 2012; Bourdon *et al.*, 2021; Stratopoulou *et al.*, 2023), and mounting evidence indicates that inflammation disrupts processes crucial for successful implantation and pregnancy maintenance (Guo *et al.*, 2018). However, most work has focused on immune changes at or after conception, leaving a gap in our understanding of pre-conception immune alterations.

Addressing this gap is crucial for identifying early pathogenic events and for pinpointing the most effective strategies to safeguard fertility. Therefore, the aim of the present study was to investigate the uterine immune environment prior to pregnancy during the development of the pathology in tamoxifen-induced adenomyosis mice. By defining the timing and nature of immune dysregulation in adenomyosis, we aim to refine our understanding of its pathogenesis and highlight potential avenues for intervention. Ultimately, pinpointing the onset of these immune disturbances may pave the way for preventive measures to counteract the reduced fertility often observed in adenomyosis.

## Materials and methods

### Mouse model of adenomyosis (experimental design)

**Figure 1.**
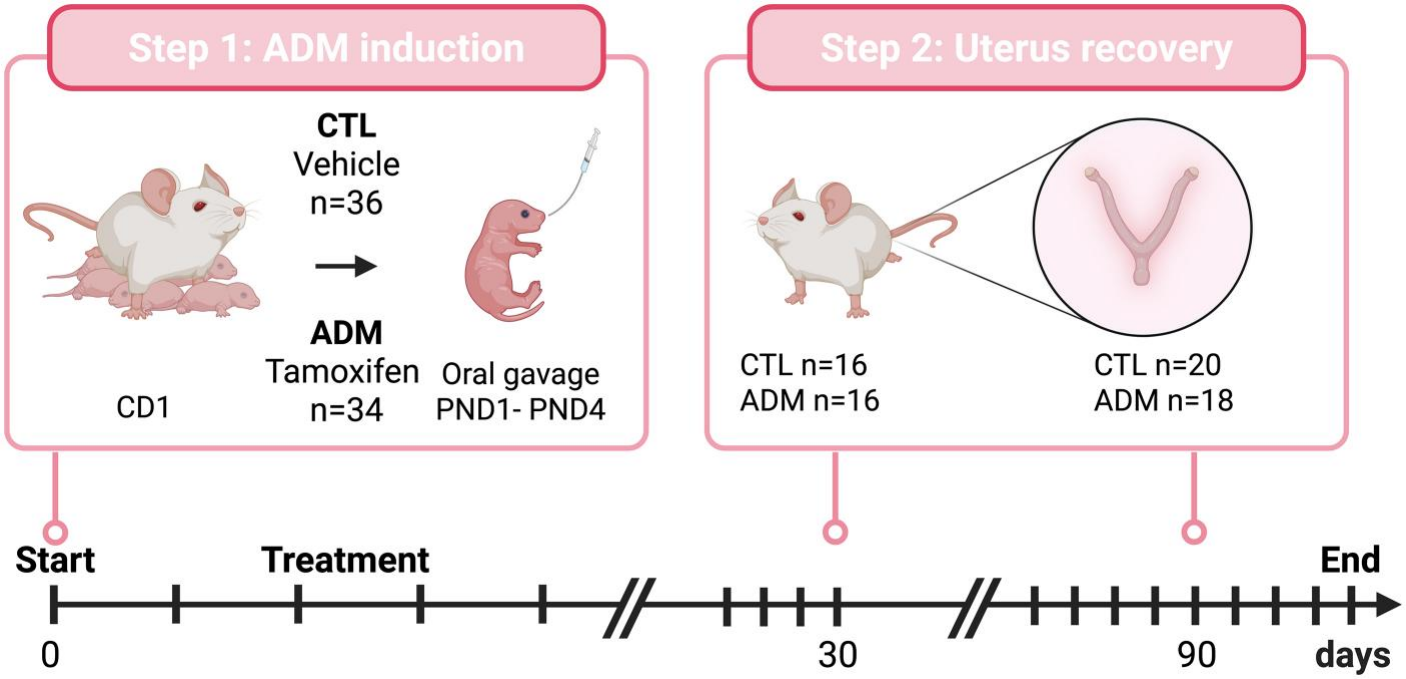
**Experimental design.** Schematic overview of the experimental design and analysis workflow in control (CTL) and adenomyosis (ADM) mice at 1- and 3-month post-induction. Created in BioRender. Munaut, C. (2025) https://BioRender.com/3l3vb9j.

Animal experiments in this study were approved by the Animal Ethics Committee of the University of Liege (approval number # 2387) and conducted following ARRIVE guidelines. In total, 70 neonatal female CD1 mice were included in the study and divided into two groups. The first group received an oral administration of vehicle from postnatal day (PND) 1–5, while the second group received an oral administration of tamoxifen (2.7 µmol/kg = 1 mg/kg) (Sigma, T5648, St Louis, MO, USA) suspended in a peanut oil/lecithin/condensed milk mixture (2:0.2:3, v/v) at a dose volume of 5 µl/g body weight. This tamoxifen treatment was used to induce adenomyosis as previously described (Green *et al.*, 2005) (Fig. 1).

All mice were housed under controlled conditions, with a temperature maintained at approximately 21°C and a 12-h light/dark cycle, with ad libitum access to food and water. Mice were sacrificed at two different time points: an early stage, 4 weeks after treatment initiation, where 16 mice per group were euthanized, and an advanced stage, 12 weeks after treatment initiation, where 20 mice from the vehicle group and 18 mice from the tamoxifen group were sacrificed (Fig. 1). These time points were selected based on our previous characterization of this model: at 1-month post-induction, mice consistently display early disruption of the myometrium without invasive adenomyotic lesions (pre-lesional stage), whereas by 3-month post-induction, adenomyotic lesions are confirmed in nearly all animals, with the majority reaching grade 3 severity (Squatrito *et al.*, 2024). These two windows thus allow capturing both the onset of uterine alterations and the established disease stage, providing a relevant framework to study temporal immune remodeling.

To minimize variability related to the estrous cycle, mice were synchronized by subcutaneous injection of 17β-estradiol (100 ng/100 ml) for three consecutive days before sacrifice, as previously described (De Clercq *et al.*, 2017).

Mice were first anesthetized with isoflurane inhalation (5% in oxygen) until loss of pedal reflex, and subsequently euthanized by cervical dislocation, in accordance with the AVMA Guidelines for the Euthanasia of Animals (Leary *et al*., 2020) and the approved institutional protocol.

Following euthanasia, uterine tissues were collected for further analysis. To ensure enough immune cells for flow cytometry analysis, one-half uterine horn from two mice were pooled. The remaining uterine horn from each mouse was divided into two (1-month-old mice) or three (3-month-old mice) parts: one portion was snap-frozen in liquid nitrogen and then stored at −80°C for RNA extraction and quantitative real-time PCR (RT-qPCR) analyses, while the other was fixed in 4% formalin and embedded in paraffin for immunohistochemistry (IHC) analyses.

### Histochemical analysis

To confirm the presence or absence of adenomyotic lesions, double immunofluorescence staining was performed on uterine sections using αSMA-FITC (Sigma, F3777, 1/400, St Louis, MO, USA) and EpCAM (Cell Signaling, 93790, 1/400, Danvers, MA, USA) antibodies. αSMA antibody was directly conjugated to FITC, allowing visualization of the myometrium in green, while EpCAM was detected using a tyramide amplification system conjugated to Cy3, marking the epithelial glands in red.

Briefly, paraffin-embedded uterine sections were first deparaffinized and rehydrated, followed by antigen retrieval using an autoclave (11 min, 126°C, 1.3 bar). After cooling for 20 min at room temperature (RT), endogenous peroxidase activity was blocked by incubating the sections in 3% hydrogen peroxide for 20 min at RT. To prevent non-specific binding, sections were incubated for 20 min at RT in the ‘Animal-Free Blocking Solution’ (Cell Signaling).

Primary antibodies were diluted in REAL antibody diluent (Dako, Santa Clara, CA, USA) and incubated for 1 h at RT. After primary antibody incubation, sections were incubated with a secondary antibody conjugated to horseradish peroxidase (EnVision/HRP, Dako) for 30 min at RT. The revelation of EpCAM was performed using a tyramide amplification system conjugated to Cy3 (PerkinElmer, Shelton, CT, USA), allowing visualization of the epithelial glands in red. Sections were then mounted using DAPI Fluoromount-G mounting medium (SouthernBiotech, Birmingham, AL, USA).

Fluorescently labeled sections were scanned using the SLIDEVIEW VS200 research slide scanner (Olympus, Antwerp, Belgium) equipped with a Uplan-XApo 20× 0.8 NA objective (Olympus).

### Flow cytometry analysis

Uterine tissue fragments from CD1 mice were placed in a digestion solution composed of Hank’s balanced salt solution (HBSS) and collagenase type Ia (2.5 mg/ml) and incubated at 37°C with agitation for 1 h. To facilitate tissue dissociation, pipetting (40 up-and-down motions with a P1000) was performed every 15 min. Following digestion, EDTA (1:100) was added, and additional pipetting was performed for 2 min to ensure complete tissue dissociation. The resulting cell suspension was filtered through a 70 µm mesh and centrifuged at 310*g* for 10 min at 4°C. The supernatant was discarded, and the pellet was resuspended in FACS buffer (HBSS supplemented with 2% FBS and 1% penicillin/streptomycin). To prevent non-specific binding, cells were incubated for 20 min at 4°C with a blocking solution (Fc block; 1:500 dilution in HBSS) prior to antibody staining. A mixture of primary antibodies, targeting various cell populations (Table 1), was prepared in FACS buffer. Each sample was incubated with appropriate dilution of antibody mix in the dark, on ice, for 30 min, with gentle vortexing every 10 min. After incubation, cells were washed with FACS buffer and centrifuged at 250*g* for 5 min at 4°C. To exclude dead cells, a viability dye (Zombie Aqua) was used according to the manufacturer’s instructions (Biolegend, San Diego, CA, USA). To correct for spectral overlap between fluorophores, a compensation matrix was generated using single-stained beads labeled with individual fluorochromes. Fluorescence minus one (FMO) controls were systematically included for key markers to define gating thresholds. A uniform gating strategy was applied consistently across all samples within each experimental group, ensuring reliable discrimination of positive and negative subsets for each immune population. After staining, samples were filtered through 40 µm filters into fresh FACS tubes prior to analysis.

**Table 1.** Antibodies and viability dye used for flow cytometric analysis of mouse uterine immune cells.

Target (antigen)	Fluorochrome	Company	**City**	**Country**	Catalogue No.
CD45	PeCy7	BioLegend	San Diego, CA	USA	103114
F4/80	BV605	BioLegend	San Diego, CA	USA	123133
CD11b	BV785	BioLegend	San Diego, CA	USA	101243
CD86	Pe-Dazzle594	BioLegend	San Diego, CA	USA	105041
CD163	APC	BioLegend	San Diego, CA	USA	156705
CD19	FITC	BioLegend	San Diego, CA	USA	152404
CD3	BB700	BD Biosciences	San Jose, CA	USA	742175
NKp46	BV650	BioLegend	San Diego, CA	USA	137635
CD4	BV711	BioLegend	San Diego, CA	USA	100447
CD8	APC-Cy7	BD Biosciences	San Jose, CA	USA	561967
Live/dead fixable aqua	—	Thermo Fisher Scientific	Waltham, MA	USA	L34957
Fc block	—	BD biosciences	San Jose, CA	USA	553142

Cell sorting was performed using an Aria III flow cytometer (BD Biosciences, San Jose, CA, USA), with all available cells from each sample being analyzed and sorted to maximize recovery of the targeted immune cell populations. Specifically, M1- and M2-like macrophage subsets were sorted with the initial aim of performing downstream RT-qPCR analyses to further characterize their polarization profiles. However, due to the low number of recovered cells in several samples, these downstream assays could not be reliably performed, and the present work therefore focuses on the flow cytometry-based characterization of the uterine immune microenvironment. One pooled sample (two uteri) was excluded from the analysis because the total number of recovered cells was abnormally low (<30 000 cells), whereas all other samples yielded a minimum of 600 000 cells analyzed. Flow cytometry data were analyzed using FlowJo software (version 10, Ashland, OR, USA). The gating strategies used for the flow cytometry analysis are shown in Supplementary Fig. S1.

### Real-time PCR analysis

Gene expression was assessed by RT-qPCR. Total RNA was extracted from individual samples using the RNeasy Mini kit (Qiagen, Hilden, Germany) following the manufacturer’s instructions.

For each sample, 1 µg of total RNA was reverse transcribed into complementary DNA (cDNA) using the FastGene Scriptase II Ready Mix (Nippon Genetics, Düren, Germany). RT-qPCR was performed using SYBR Green PCR Master Mix and specific primers (Table 2) on a QuantStudio 3 Real-Time PCR System (Thermo Fisher Scientific, Waltham, MA, USA).

**Table 2.** Primers for RT-qPCR.

Gene	Forward primer (5′–3′)	Reverse primer (5′–3′)	Amplicon size (bp)
*Il6*	ACCTGGAGTACATGAAGAACAACTT	GCTCTTGGTTGAAGATATGAATTAGA	102
*Il6r*	TGCAGTTCCAGCTTCGATACCG	TGCTTCACTCCTCGCAAGGCAT	112
*Il10*	GCTCTTACTGACTGGCATGAG	CGCAGCTCTAGGAGCATGTG	105
*Stat3*	CAATACCATTGACCTGCCGAT	GAGCGACTCAAACTGCCCT	109
*Ptgs2*	GCGACATACTCAAGCAGGAGCA	AGTGGTAACCGCTCAGGTGTTG	132

Gene expression levels were analyzed using the 2^−ΔΔCt^ method, with all samples measured in duplicate. To minimize variability related to the estrous cycle, only mice in estrus were selected for RT-qPCR analyses, since the expression of several genes, including inflammation-related genes, may fluctuate depending on cycle stage. To normalize gene expression, *Rplp0* and *Gapdh* were used as housekeeping genes, as recommended by Lin *et al.* for gene expression studies in the mouse uterus (Lin *et al.*, 2013).

### Statistical analysis

All statistical analyses were performed using GraphPad Prism (version 9.0, San Diego, CA, USA).

For mouse and uterine weights, data were normally distributed, as assessed by the Shapiro–Wilk test and visual inspection of QQ plots. Therefore, results are presented as mean ± SD, and parametric unpaired two-tailed *t*-tests were used to compare groups.

For flow cytometry (FACS) and RT-qPCR analyses, the non-parametric Mann–Whitney *U* test (two-tailed) was applied. In these cases, results are presented as medians (interquartile ranges).

Exact *P*-values are reported in the text. A *P*-value was considered statistically significant when *P* < 0.05. The number of replicates (biological or technical) is specified in each figure legend.

## Results

### Development of adenomyosis after neonatal tamoxifen treatment

To assess adenomyosis progression, we analyzed uterine morphology in control (CTL) and tamoxifen-treated (ADM) mice at 1- and 3-month post-treatment.

At both time points, none of the control mice exhibited signs of adenomyosis, confirming that the condition does not spontaneously develop in ‘young’ untreated animals. In contrast, uteri from ADM mice sacrificed 30-day post-treatment displayed early signs of myometrial remodeling, characterized by tissue disorganization and structural alterations. However, in most cases, these modifications had not yet progressed into fully developed adenomyotic lesions, suggesting an initial phase of disease establishment without substantial glandular invasion.

**Figure 2.**
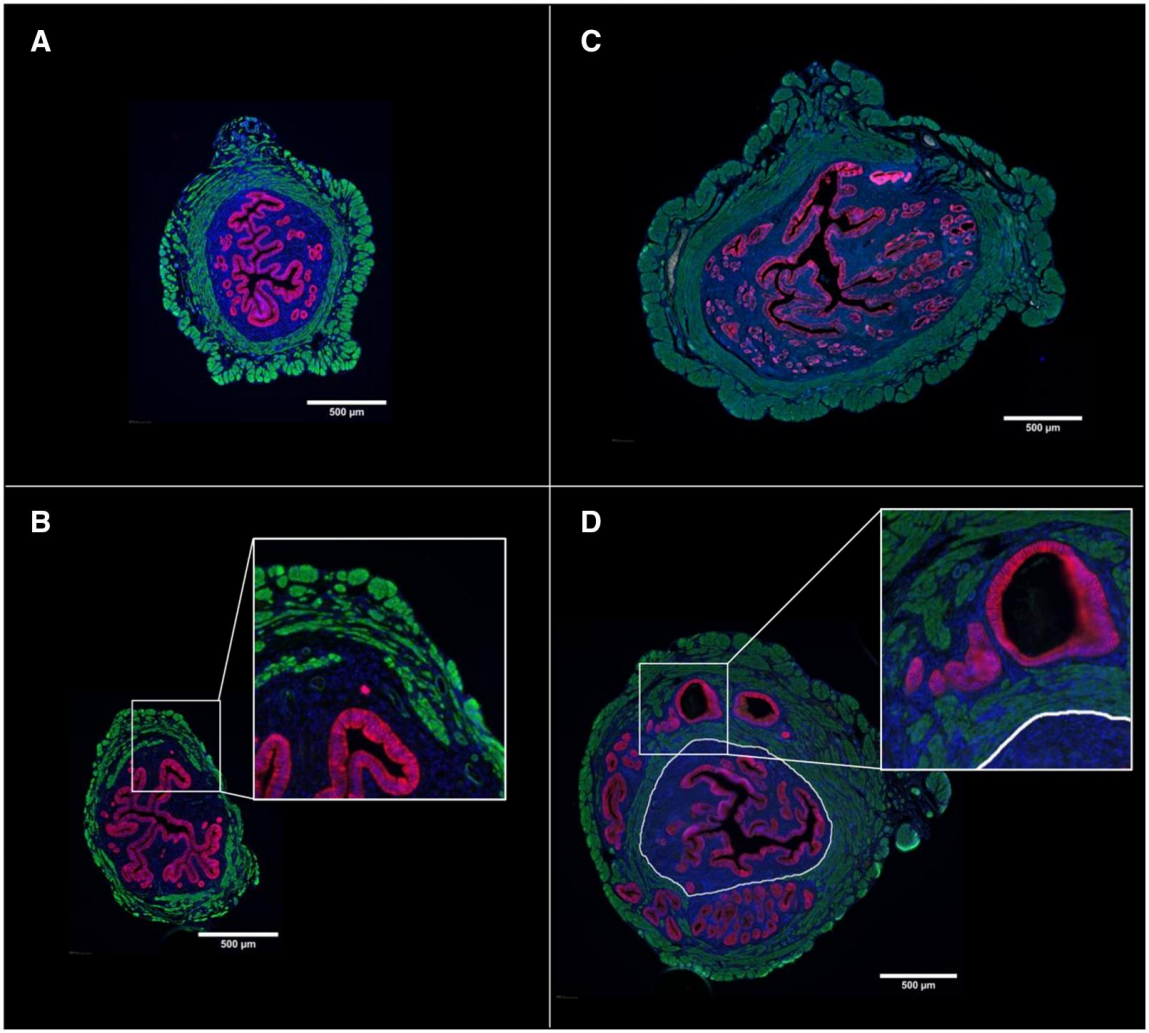
**Uterine morphology in control (CTL) and adenomyosis (ADM) mice.** Representative immunofluorescence staining of transverse uterine sections at 1 and 3 months. aSMA (green) marks the myometrium, EpCAM (red) labels epithelial glands, and DAPI (blue) stains cell nuclei. At 1 month, CTL uteri display normal morphology (**A**), whereas ADM uteri show disrupted myometrium (**B**, higher magnification). At 3 months, CTL uteri remain normal (**C**), while ADM uteri present adenomyotic lesions (**D**, higher magnification). In (D), the myometrium–endometrium boundary is delineated (white circle). Scale bar, 500 µm.

By 90-day post-treatment, ADM mice exhibited prominent adenomyosis lesions, with the majority classified as grade 3, consistent with previously published data (Squatrito *et al.*, 2024). This progressive histopathological transformation supports the hypothesis that neonatal tamoxifen exposure induces sustained uterine alterations that gradually lead to advanced adenomyosis (Fig. 2).

In addition to morphological changes, physiological parameters were also affected. At 1-month post-treatment, body weight was significantly lower in ADM mice (22.86 ± 2.34 g) compared to controls (24.71 ± 1.55 g; *P *= 0.0133). However, this difference was no longer observed at 3 months (ADM: 34.98 ± 5.00 g vs CTL: 33.52 ± 3.45 g; n.s.), suggesting a transient growth delay. In contrast, uterine weight remained significantly reduced in ADM mice at both time points. At 1 month, ADM mice exhibited a dramatic reduction (0.036 ± 0.013 g) compared to controls (0.096 ± 0.010 g; *P *< 0.0001). A significant difference persisted at 3 months (ADM: 0.093 ± 0.036 g vs CTL: 0.145 ± 0.044 g; *P *= 0.0004), indicating sustained disruption of uterine homeostasis following neonatal tamoxifen exposure. Consistently, the uterine weight-to-body weight ratio was also significantly lower in ADM compared to CTL mice at both 1 month (ADM: 0.0016 ± 0.0006 vs CTL: 0.004 ± 0.0004; *P* < 0.0001) and 3 months (ADM: 0.0028 ± 0.001 vs CTL: 0.0044 ± 0.001; *P *= 0.0002), further confirming impaired uterine development (Table 3).

**Table 3.** Body and uterine weights in control (CTL) and tamoxifen-treated (ADM) mice at 1- and 3-month post-treatment.

Weight (g)	1 month			3 months		
	CTL (n = 16)	ADM (n = 16)	*P*	CTL (n = 20)	ADM (n = 18)	*P*
**Body (B)**	24.71 ± 1.55	22.86 ± 2.34	*	33.52 ± 3.45	34.98 ± 5.00	n.s.
**Uterus (U)**	0.096 ± 0.010	0.036 ± 0.013	****	0.145 ± 0.044	0.093 ± 0.035	***
**Ratio U/B**	0.004 ± 0.0004	0.0016 ± 0.0006	****	0.0044 ± 0.001	0.0028 ± 0.001	***

Data are expressed as mean ± standard deviation (SD). Statistical significance was assessed using unpaired two-tailed *t*-tests.

* *P *< 0.05, *** *P* < 0.001, **** *P *< 0.0001; n.s. = not significant.

Together, these findings provide insight into the temporal dynamic of adenomyosis development following neonatal tamoxifen exposure and highlight key histopathological and physiological alterations that precede the establishment of severe disease.

### Temporal modulation of innate and adaptive immune responses in a mouse model of adenomyosis

Adenomyosis induces profound modulation of the uterine immune microenvironment, a phenomenon suspected to impair fertility. To characterize these immunological alterations, we profiled innate and adaptive immune cell subsets in uteri from CTL and ADM mice at 1 month (early stage) and 3 months (advanced stage) post-induction. Multiparametric flow cytometry enabled precise quantification of each subset and revealed time-dependent alterations in both innate and adaptive immune compartments, as detailed below.

#### Modulation of macrophages and other innate immune subsets in the adenomyotic uterus

##### NK cells

**Figure 3.**
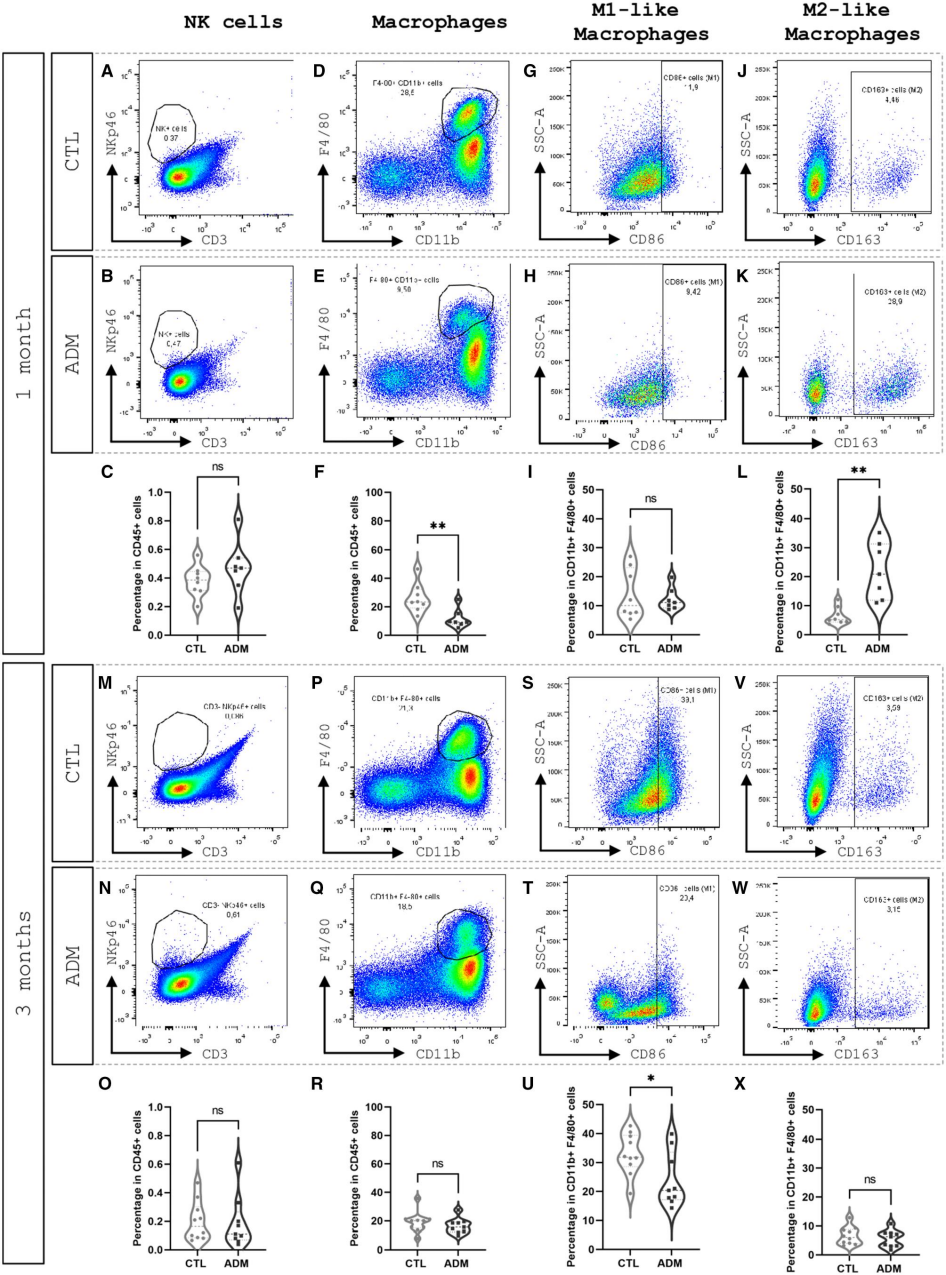
**Innate immune cells in control (CTL) and adenomyosis (ADM) uteri.** Representative flow cytometry dot plots and violin plots showing the percentage of natural killer (NK) cells (CD3^−^ NKp46^+^) (**A–C**, **M–O**), total macrophages (CD11b^+^ F4/80^+^) (**D–F**, **P–R**), M1-like macrophages (CD11b^+^ F4/80^+^ CD86^+^) (**G–I**, **S–U**), and M2-like macrophages (CD11b^+^ F4/80^+^ CD163^+^) (**J–L**, **V–X**) at 1 and 3 months. Adenomyosis induced an early reduction of total macrophages with an increase in the M2-like subset, followed by a decrease in M1-like macrophages 3-month post-tamoxifen treatment; NK-cell frequencies remained unchanged across time points. Sample sizes: 1 month (CTL 16 mice pooled (n = 8); ADM 16 mice pooled with one exclusion (n = 7)); 3 months (CTL 20 mice pooled (n = 10); ADM 18 mice pooled (n = 9)). Statistical significance was determined using the Mann–Whitney *U* test: *P < 0.05, **P < 0.01; ns, not significant.

At both early (1 month) and advanced (3 months) stages, the proportion of uterine NK cells (CD3^−^ NKp46^+^) remained comparable between ADM and CTL mice (Fig. 3A–C, M–O), with no statistically significant differences (*P* = 0.2440 and *P* = 0.237, respectively). These findings suggest that adenomyosis does not substantially impact NK-cell-mediated immune surveillance at either time point.

##### Macrophages and subtypes

At the early stage, the proportion of total macrophages (CD11b^+^ F4/80^+^) was significantly reduced in ADM mice compared to CTL (median 9.27% vs 23.35%, *P* = 0.0093; Fig. 3D–F). This reduction was accompanied by a marked shift toward an increased frequency of M2-like macrophages (CD11b^+^ F4/80^+^ CD163^+^) in ADM mice (median 20.9% vs 5.18%, *P* = 0.0012, Fig. 2G–L), while M1-like macrophages (CD11b^+^ F4/80^+^ CD86^+^) remained comparable between groups.

At the advanced stage, the total macrophage population was no longer significantly different between ADM and CTL mice (median 15.70% vs 20.05%, *P* = 0.189, Fig. 3P–R). However, a significant decrease in M1-like macrophages was observed in the uteri of ADM mice compared to CTL mice (median 20.40% vs 31.85%, *P* = 0.035, Fig. 3S–U), whereas M2-like macrophages remained similar between groups (Fig. 3V–X).

Together, these results demonstrate a time-dependent modulation of macrophage polarization in ADM mice, with a significant reduction of M1-like macrophages and relatively stable levels of M2-like cells. This evolving macrophage profile is consistent with a progressive shift toward an anti-inflammatory immune environment in the adenomyotic uterus.

#### Changes in adaptive immune cells

##### B cells

**Figure 4.**
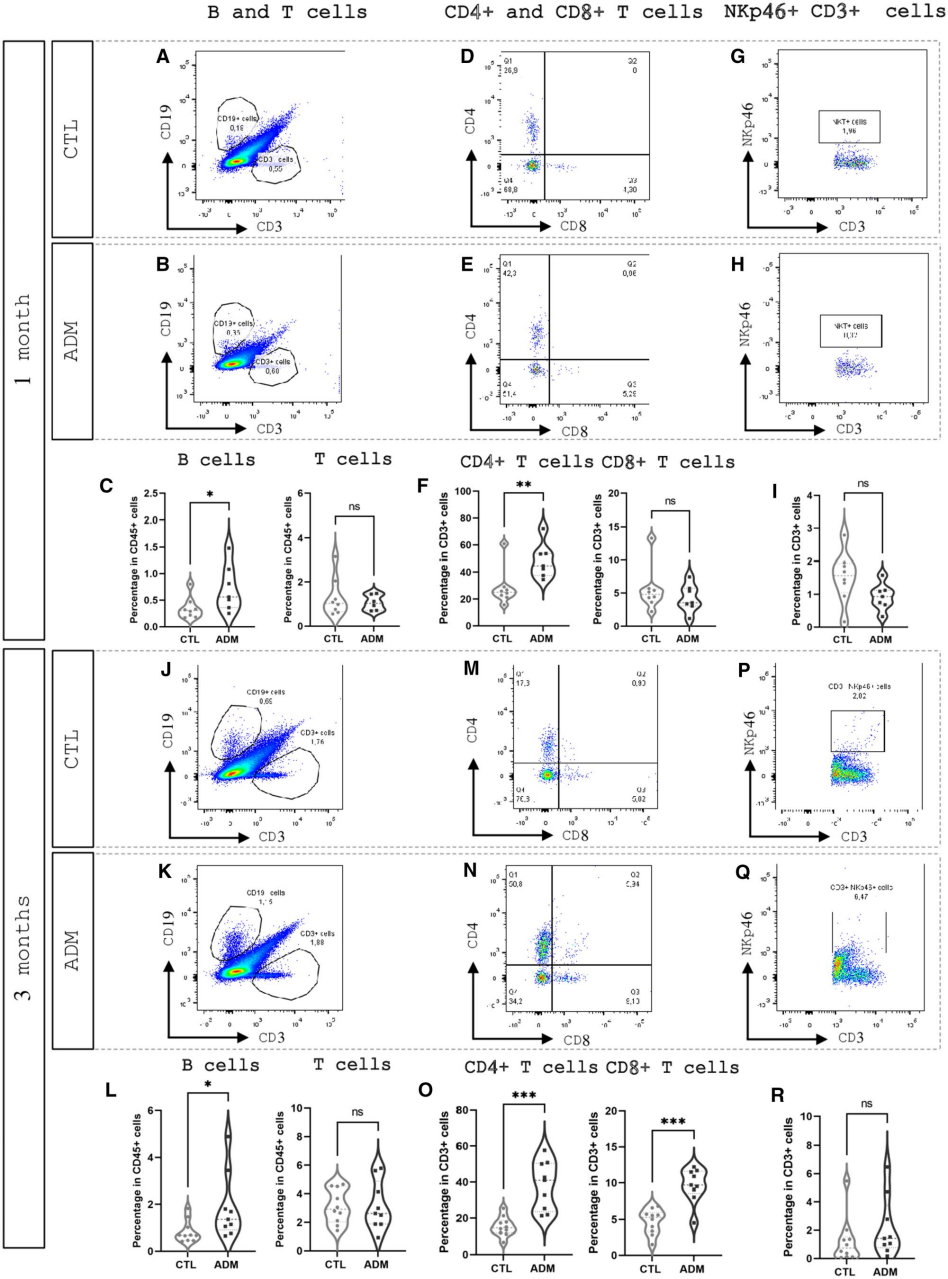
**Adaptive immune cells in control (CTL) and adenomyosis (ADM) uteri.** Representative flow cytometry dot plots and violin plots showing the percentage of B cells (CD19^+^) and total T cells (CD3^+^) (**A–C**, **J–L**), helper T cells (CD4^+^) and cytotoxic T cells (CD8^+^) (**D–F**, **M–O**), and NKT cells (NKp46^+^ CD3^+^) (**G–I**, **P–R**) at 1 and 3 months. ADM uteri showed increased B-cell percentage and higher CD4^+^ T-cell levels at both time points, while CD8^+^ T cells increased only 3-month post-tamoxifen treatment; total T and NKT cells remained unchanged. Sample sizes: 1 month (CTL 16 mice pooled (n = 8); ADM 16 mice pooled with one exclusion (n = 7)); 3 months (CTL 20 mice pooled (n = 10); ADM 18 mice pooled (n = 9)). Statistical significance was determined using the Mann–Whitney *U* test: *P < 0.05, ***P < 0.001; ns, not significant.

At the early stage (1 month), the proportion of uterine B cells (CD3^−^CD19^+^) was significantly increased in ADM mice compared to CTL (median 0.56% vs 0.30%, *P* = 0.029). This elevation persisted at the advanced stage (3 months), with B-cell frequencies remaining significantly higher in ADM mice (median 1.36% vs 0.68%, *P* = 0.026, Fig. 4A–C, J–L). These findings indicate a sustained enrichment of B cells in the adenomyotic uterus, suggesting a long-lasting modulation of the humoral immune response in this pathological context.

##### T cells and subtypes

We first quantified total T lymphocytes (CD3^+^CD19^−^) in uteri from CTL and ADM mice at 1 month (early stage) and 3 months (advanced stage) post-induction. The overall proportion of CD3^+^ T cells did not differ significantly between ADM and CTL groups at either time point, indicating that adenomyosis does not affect total T-cell infiltration (Fig. 4A–C, J–L).

**Figure 5.**
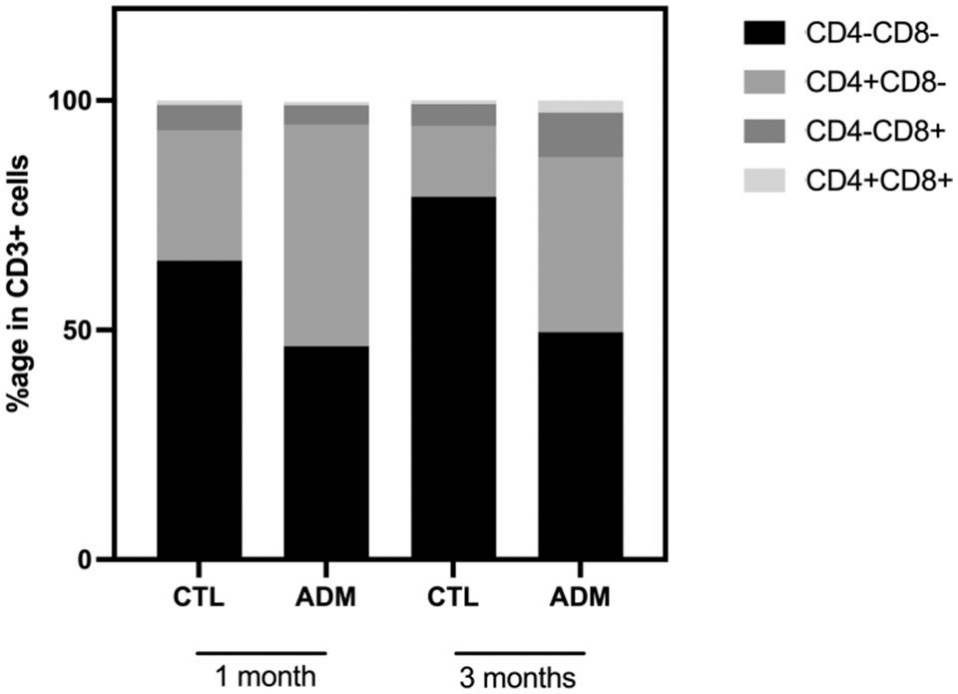
**Distribution of uterine CD3^+^ T-cell subsets.** Relative distribution of CD4^−^CD8^−^ (double-negative, DN), CD4^+^CD8^−^, CD4^−^CD8^+^, and CD4^+^CD8^+^ (double-positive, DP) T-cell subsets within the CD3^+^ compartment at 1 and 3 months. ADM uteri showed a reduction of DN T cells and increased CD4^+^ single-positive cells at both time points, while CD8^+^ single-positive and DP T cells increased only 3-month post-tamoxifen treatment. Each bar represents the mean of individual percentages. Sample sizes: 1 month, CTL (16 pooled, n = 8), ADM (16 pooled, one exclusion, n = 7); 3 months, CTL (20 pooled, n = 10), ADM (18 pooled, n = 9). ADM, adenomyosis; CTL, control.

We then analyzed the distribution of four CD3^+^ T-cell subsets based on CD4 and CD8 expression: double-negative (DN, CD4^−^CD8^−^), CD4 single-positive (CD4^+^CD8^−^), CD8 single-positive (CD4^−^CD8^+^), and double-positive (CD4^+^CD8^+^) cells (Figs 4D–F, M–O and 5).

Across all groups, DN T cells represented the predominant subset, as shown by the 100% stacked bar graph (Fig. 5). However, this population was significantly reduced in ADM mice compared to CTL both at 1 month (median 51.4% vs 68.85%; *P *= 0.0084) and at 3 months (median 47.3% vs 80.6%; *P *< 0.0001, Table 4). Conversely, the frequency of CD4^+^CD8^−^ T cells was significantly increased in ADM mice at both time points (1 month: *P *= 0.0093; 3 months: *P* = 0.0002), as was the proportion of CD4^+^CD8^+^ double-positive cells (1 month: *P *= 0.5335; 3 months: *P *= 0.0128, Table 4). No significant difference was observed in the CD8^+^CD4^−^ subset at 1 month (*P *= 0.5358), although a moderate increase was detected at 3 months in ADM mice (*P *= 0.0006).

**Table 4.** Quantitative comparison of CD3^+^ T-cell subsets in the murine uterus at 1- and 3-month post-induction.

Time	Group	CD4^−^CD8^−^ (IQR)	CD4^+^CD8^−^ (IQR)	CD4^−^CD8^+^ (IQR)	CD4^+^CD8^+^ (IQR)
1 month	CTL	68.85 (66.55–70.50)	25.15 (21.63–28.55)	4.80 (3.78–5.61)	0.48 (0.41–1.48)
	ADM	51.40** (38.8–8.30)	44.70** (37.60–53.50	3.54 (3.04–5.70)	0.56 (0.47–1.35)
3 months	CTL	80.60 (74.08–82.53)	14.6 (11.83–18.68)	5.28 (3.25–5.95)	0.53 (0.25–1.00)
	ADM	47.30**** (38.40–60.95)	41.00*** (23.55–50.45)	9.70*** (8.58–11.60)	1.96* (1.12–4.25)

Median values (% of CD3^+^) with interquartile ranges (IQR) are presented. Statistical comparisons were performed using the Mann–Whitney *U* test between control (CTL) and adenomyosis (ADM) groups at each timepoint.

* *P* < 0.05,

** *P* < 0.01,

*** *P* < 0.001,

**** *P* < 0.0001.

In addition, natural killer T cells (NKT, CD3^+^ NKp46^+^), a unique population bridging innate and adaptive immunity through rapid cytokine secretion, were also assessed. Their frequency remained low and did not differ significantly between ADM and CTL groups at either time point, suggesting that NKT cell involvement in the uterine immune response to adenomyosis is limited under these conditions.

### Expression of *Il6*, *Il6R*, *Il10*, *Stat3*, and *Ptgs2* mRNA in control and adenomyotic uteri

**Figure 6.**
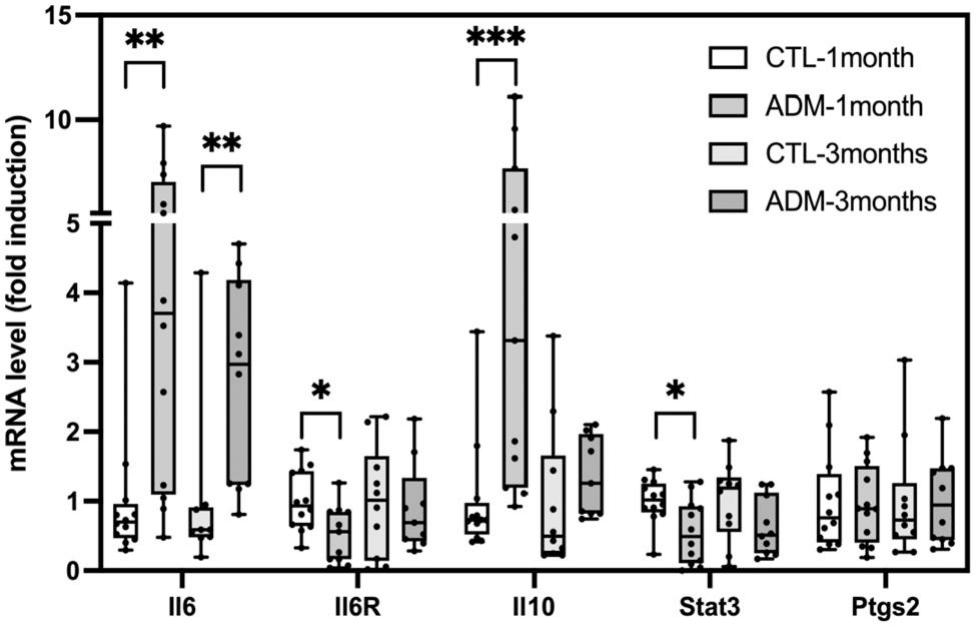
**Expression levels of inflammatory and immunoregulatory transcripts.** Relative mRNA expression of *Il6*, *Il6r*, *Il10*, *Stat3*, and *Ptgs2* in uterine tissues of control (CTL) and adenomyosis (ADM) mice at 1 and 3 months (RT-qPCR). At 1 month, ADM uteri showed increased expression of *Il6* and *Il10* with decreased expression of *Il6r* and *Stat3*, while *Ptgs2* expression remained unchanged; at 3 months, only *Il6* expression remained elevated. Bars represent median with interquartile range. Sample sizes: 1 month, CTL and ADM n = 12; 3 months, CTL and ADM n = 10. *P*-values were calculated using the Mann–Whitney *U* test: *P < 0.05, **P < 0.01, ***P < 0.001.

To assess the cytokine and signaling profile associated with the altered immune landscape in adenomyotic uteri, we measured *Il6*, *Il6R*, *Il10*, *Stat3*, and *Ptgs2* mRNA levels by RT-qPCR in CTL and ADM mice at 1- and 3-month post-induction (Fig. 6).

At 1 month, *Il6* and *Il10* transcripts were significantly upregulated in ADM uteri compared to CTL (*Il6: P* = 0.0014; *Il10: P* = 0.0003), whereas *Il6R* and *Stat3* expression were significantly downregulated (*IL6R: P* = 0.0132; *Stat3: P* = 0.0205). In contrast, *Ptgs2* levels remained unchanged (*P* > 0.9999). At 3 months, *Il6* remained elevated in ADM mice (*Il6: P* = 0.003), but *Il6R*, *Stat3* and *Il10* expression were no longer different from CTL (*P* = 0.7759; *P* = 0.1431; *P* = 0.1564), and *Ptgs2* still showed no significant change (*P* = 0.7959).

Together, these data reveal a dysregulated cytokine signaling in adenomyotic uteri, marked by sustained *Il6* and *Il10* induction despite transient *Stat3* repression and no alteration of *Ptgs2* expression.

## Discussion

### General overview of immune remodeling

In this study, we investigated the temporal remodeling of the uterine immune microenvironment in a murine model of adenomyosis, integrating cellular and molecular analyses. Although flow cytometry was performed prior to gene expression analysis, the following overview synthesizes both datasets to provide a comprehensive picture of immune dynamics.

Our data revealed coordinated alterations in both innate and adaptive compartments. Early adenomyosis was characterized by reduced macrophage numbers with a skewing toward M2-like polarization, while later stages showed selective depletion of M1-like macrophages. In parallel, B cells were consistently elevated, and CD3^+^ T cells, although stable in total numbers, underwent a marked redistribution across subsets. At the molecular level, *Il6* mRNA was persistently upregulated, while *Il10* showed only transient elevation, and *Ptgs2* remained unchanged. Collectively, these findings suggest that adenomyosis provoked a progressive disruption of the innate–adaptive balance, shifting from an early compensatory anti-inflammatory response to a state of unresolved inflammation that may impair uterine homeostasis.

### Time-dependent redistribution of CD3^+^ T-cell subsets

Flow cytometry revealed a pronounced reshaping of CD3^+^ T-cell subsets in the ADM uterus. The proportion of double-negative (CD4^−^CD8^−^) cells significantly declined at both 1 and 3 months, consistent with a progressive depletion of this subset. In parallel, CD4^+^ T cells increased significantly at both timepoints, suggesting early activation of helper T-cell responses. CD8^+^ T cells showed a delayed but robust expansion at 3 months, indicative of cytotoxic effector engagement.

Notably, CD4^+^CD8^+^ double-positive T cells emerged significantly at 3 months. These cells, typically rare in peripheral tissues, may represent transitional or activated phenotypes associated with chronic immune stimulation (Parel and Chizzolini, 2004; Hagen *et al.*, 2023). This time-dependent redistribution of T-cell subsets coincides with transient *Stat3* downregulation, suggesting that dampened STAT3 signaling may influence lineage commitment toward CD4^+^ and CD8^+^ effector functions. Similar alterations affecting CD8^+^ T cells have been reported in the endometrium of patients with adenomyosis and/or endometriosis, highlighting changes in cytotoxic T-cell populations associated with disease severity (Kisovar *et al.*, 2023; Liu *et al.*, 2023). Together, these data support the hypothesis of an evolving adaptive immune response in adenomyosis, potentially contributing to lesion persistence and fertility impairment.

### Physiological correlates of immune dysregulation

In addition to immune remodeling, we also observed significant physiological alterations. ADM mice exhibited a transient reduction in body weight at 1-month post-induction, which normalized by 3 months. This early growth delay may reflect systemic effects of neonatal exposure or early inflammatory stress. More strikingly, uterine weight remained significantly reduced in ADM mice at both 1 and 3 months, suggesting a sustained disruption of uterine growth and tissue homeostasis. These changes parallel our molecular findings and support the idea that chronic immune imbalance may impair uterine remodeling, even beyond the acute lesion phase. These physiological observations are consistent with previous studies using neonatal tamoxifen to induce adenomyosis. Several reports have shown that uterine weight is significantly reduced in tamoxifen-treated mice at both early and late timepoints, even when body weight is unaffected (Parrott *et al.*, 2001; Green *et al.*, 2005). Our observation suggests that neonatal tamoxifen exposure disrupts uterine growth more specifically than systemic development.

Together, these immunological and physiological alterations underscore a coordinated disruption of uterine homeostasis in adenomyosis, prompting us to consider the underlying mechanisms by which IL6–driven inflammation and STAT3 modulation impair tissue growth and remodeling.

### Macrophage polarization and IL-6/IL-10 imbalance

Our data suggest that early innate changes establish the inflammatory context for adaptive responses. The reduction in macrophage numbers paired with M2 polarization may hinder clearance of ectopic endometrial cells while promoting tissue remodeling and fibrosis (Gordon, 2003). Loss of M1‐like macrophages 3 months after adenomyosis induction may further diminish classical inflammatory functions necessary for lesion resolution (Ota *et al.*, 1998; Riccio *et al.*, 2018).

Paralleling endometriosis literature—where M2‐polarized macrophages accumulate in lesions and promote tissue invasion (Bacci *et al.*, 2009; Nie *et al.*, 2018), while studies on M1/M2 prevalence yield mixed findings (Itoh *et al.*, 2013; Takebayashi *et al.*, 2015)—our adenomyosis model similarly exhibits an early M2 dominance followed by M1 loss. This dualistic concept has recently been challenged: macrophages can undergo ‘trained immunity’ and display hybrid M1/M2 signatures that defy a simple polarization framework (Martinez and Gordon, 2014). These observations are consistent with human studies showing accumulation of M2 macrophages in adenomyotic lesions, where they contribute to tissue remodeling and lesion invasiveness (Stratopoulou *et al.*, 2023).

These macrophage dynamics appear to be closely tied to evolving cytokine environment. M1-like macrophages are known sources of pro-inflammatory cytokines such as IL-6, whereas M2-like macrophages secrete anti-inflammatory mediators like IL-10 (Martinez and Gordon, 2014). At 1 month, we observed increased M2-like macrophages and elevated *Il10* mRNA, suggesting an early anti-inflammatory response. However, *Il6* was already significantly upregulated despite unchanged M1-like macrophage numbers, implying non-immune sources such as stromal or epithelial cells may contribute. By 3 months, M1-like macrophages were reduced and *Il10* levels normalized, whereas *Il6* remained elevated, pointing to a shift toward chronic, unresolved inflammation in the ADM uterus. Elevated *IL6* and altered *IL10* dynamics have also been reported in human endometrial tissues from patients with adenomyosis, supporting the translational relevance of these cytokine patterns, which are known to influence endometrial receptivity and implantation success (Zhihong *et al.*, 2016; Wang *et al.*, 2018).

These local and systemic immune changes have been associated with impaired fertility in a murine adenomyosis model, coinciding with alterations in local and systemic immune cells observed during the implantation window (Bourdon *et al.*, 2024).

### IL-6/STAT3 axis: chronic inflammation and therapeutic targeting

Our observation of a sustained increase in *Il6* mRNA in ADM uteri at both 1- and 3-month post-adenomyosis induction aligns with clinical reports of elevated IL-6 in the peritoneal fluid and lesional endometrium of patients with adenomyosis and endometriosis (Incognito *et al.*, 2023; Cao *et al.*, 2025). IL-6 is a pleiotropic cytokine that drives chronic inflammation by promoting STAT3 phosphorylation, leukocyte recruitment, angiogenesis, and stromal cell proliferation (Tanaka *et al.*, 2014; West, 2019).

As an activator of the JAK–STAT signaling pathway, IL-6 can act in both a pro-inflammatory and anti-inflammatory manner. Deregulation of IL-6 production can cause severe disease, such as rheumatoid arthritis, psoriasis, arteriosclerosis, and cancer. Therapeutically, monoclonal antibodies targeting IL-6 (e.g. tocilizumab) or JAK inhibitors (e.g. ruxolitinib) have already been tested in other inflammatory diseases and may represent candidate agents for repurposing in adenomyosis models (Taskin *et al.*, 2016; El-Zayadi *et al.*, 2020; Li *et al.*, 2025; Parisi *et al.*, 2025). Furthermore, accumulating evidence shows that IL-6 can interact with WNT/β-catenin signaling. For example, hypoxia-induced IL-6 secretion can activate WNT signaling in rat mesenchymal stem cells (MSCs) through the JAK2/STAT3 signaling (Mao *et al.*, 2019; Chen *et al.*, 2024). Although we did not detect sustained *Stat3* mRNA elevation—likely due to negative feedback by SOCS3 (Gao *et al.*, 2018)—the persistent *Il6* mRNA overexpression suggests that post-transcriptional activation of STAT3 or alternative pathways (e.g. MAPK) may underlie lesion progression in adenomyosis. Interestingly, both *Stat3* and *Il6r* mRNA levels were significantly reduced in ADM uteri at 1 month but returned to baseline by 3 months. This coordinated downregulation of receptor and downstream effector suggests a transient suppression of IL-6 signaling, potentially as a homeostatic brake against acute inflammatory activation. The latter normalization may reflect a loss of this regulation, allowing sustained IL-6 activity to drive chronic tissue remodeling. Although we did not directly assess regulatory T‐cell (Treg) populations—a limitation of our work—it is plausible that reduced STAT3 signaling also impairs Treg maintenance. Future studies should include FoxP3^+^ cell quantification to determine whether Treg deficiency contributes to the skewing toward CD4^+^ and CD8^+^ effector phenotypes observed in our adenomyosis model.

### Anti-inflammatory feedback and the IL-10 paradox

Concomitant with lL-6 upregulation, we detected a transient rise in *Il10* mRNA at 1 month, which normalized by 3 months. Similar patterns have been reported in chronic endometrial inflammation models, where initial IL-10 increases serve to limit tissue damage (Yang *et al.*, 2017; Park *et al.*, 2025). IL-10, produced primarily by M2 macrophages and regulatory T cells, suppresses pro-inflammatory cytokine secretion and downregulates antigen presentation (Moore *et al.*, 2001). In the context of adenomyosis, early IL-10 induction may represent a host attempt to counterbalance IL-6–driven inflammation and prevent excessive fibrosis. However, prolonged IL-10 signaling can also foster immunotolerance, facilitating lesion survival by inhibiting cytotoxic responses (Couper *et al.*, 2008). Thus, the dynamic IL-6/IL-10 interplay we observe likely reflects a bidirectional push–pull between inflammation and regulation that shapes disease outcome. Notably, the sustained IL-6 upregulation and shifts in CD3^+^ T-cell subset composition observed here could serve as candidate biomarkers for disease staging or monitoring if validated in patient samples. Their detection in endometrial biopsies or peripheral blood may provide minimally invasive tools for clinical stratification.

### COX-2 expression and technical considerations

In contrast to *Il6* and *Il10*, *Ptgs2* mRNA expression remained unchanged in ADM uteri at both 1 and 3 months. This finding diverges from a preclinical study using tamoxifen-induced adenomyosis in mice, where immunohistochemical analysis revealed high-level COX-2 protein expression in adenomyosis lesions, and pharmacological inhibition by selective COX-2 inhibitor celecoxib markedly reduced adenomyosis severity, including lesion depth, fibrosis, and epithelial–mesenchymal transition (EMT) markers (Jin *et al.*, 2020). The discrepancy may stem from methodological differences: our whole-uterus PCR approach likely dilutes localized expression seen in lesion-restricted IHC. These findings suggest that COX-2 may still play a functional role in adenomyosis progression, and protein-level analyses will be needed to clarify its contribution.

### Bridging murine models and clinical fertility impairment

Our current murine data both align with and expand recent work in adenomyosis. Previously, using the same murine model, we documented compromised reproductive capacity, thereby establishing a functional readout of lesion impact on fertility (Squatrito *et al.*, 2024). The immune alterations we now describe offer a mechanistic bridge: early M2 dominance and *Il10* rise may initially buffer tissue damage yet predispose to chronic inflammation, while later loss of M1‐like macrophages and skewed T-cell differentiation (linked to transient *Stat3* downregulation) could underlie the uterine dysfunction and subfertility observed in that study.

### Translational implications and future directions

These findings resonate with other studies. Indeed, Bourdon *et al.* showed that infiltrating immune cells cooperate with Notch1 signaling to drive epithelial-to-mesenchymal transition during lesion development in mice (Bourdon *et al.*, 2021). Building on this, they also found that reduced fertility in the same murine adenomyosis model is associated with an altered uterine immune profile at the implantation period (Bourdon *et al.*, 2024). Disrupted uterine NK-cell distribution and altered macrophage phenotypes have also been associated with lower pregnancy success in adenomyosis patients undergoing ART (Bourdon *et al.*, 2025). A recent study also evidenced that M2 macrophages accumulate within human adenomyotic lesions and actively promote endometrial cell invasiveness (Stratopoulou *et al.*, 2023). Collectively, these murine and human studies support a model in which dynamic innate–adaptive immune remodeling not only drives lesion progression but also translates into the fertility impairments we first characterized in our reproductive‐outcome study (Squatrito *et al.*, 2024). Whether these immune and structural changes translate into altered uterine contractility, receptivity, or implantation dynamics remains to be functionally addressed. Future *in vivo* or *ex vivo* assays, such as uterine peristalsis tracking or embryo transfer experiments, could help establish causality between immune remodeling and reproductive dysfunction.

### Strengths and limitations of the current approach

A major strength of our study is the use of a well‐validated murine adenomyosis model (Parrott *et al.*, 2001; Marquardt *et al.*, 2020) that isolates the effects of adenomyotic lesions from other uterine pathologies such as endometriosis or fibroids. We have characterized, for the first time, time‐dependent immune remodeling in the uterus, including shifts in macrophage polarization, T‐cell subsets, and cytokine expression.

Nevertheless, several limitations should be acknowledged. First, species differences and the simplified nature of the model may limit direct extrapolation to human adenomyosis, which often presents with heterogeneous lesions and coexists with endometriosis. Second, although mice were synchronized with β-estradiol, incomplete responses in some controls and disrupted cyclicity in ADM mice may have introduced residual variability, despite uniform synchronization across groups. Moreover, our analysis was restricted to two time points (1 and 3 months), which capture pre-lesional and established phases but do not resolve intermediate disease dynamics.

Technical factors should also be considered. Gene expression was assessed on whole uterine horns, which may dilute lesion-specific signals, and flow cytometry required pooling of uteri prior to histological confirmation, precluding one-to-one correlation with lesion grade. Furthermore, molecular analyses were limited to mRNA; protein-level validation and functional readouts remain to be performed.

Finally, our immune profiling was limited in resolution. Flow cytometry did not assess T‐cell activation markers (e.g. FoxP3, CD69) or cytokine production at the protein level, which may overlook important functional diversity. A finer characterization of macrophage polarization states (e.g. M2a, M2b, M2c) and CD4^+^ T-helper subtypes (e.g. Th1, Th2, Th17, Treg), as well as the inclusion of additional cytokines such as TNF-α, IFN-γ, IL-17, and TGF-β, will be essential to provide a more comprehensive picture of immune remodeling in adenomyosis.

## Conclusion

In conclusion, adenomyosis induction in mice provokes a stepwise immune remodeling, beginning with M2 macrophage dominance and culminating in adaptive T‐cell reprogramming underpinned by cytokine signaling shifts. These results identify immune alterations that may represent candidate markers for future investigation. Although intriguing, the translational relevance of these findings remains to be confirmed, and targeting IL6/STAT3 signaling should be considered a hypothesis-generating observation that requires further validation. Translating these findings to patients could enable immune‐based stratification and personalized interventions aimed at restoring uterine immune homeostasis.

## Supplementary Material

gaaf057_Supplementary_Data

## Data Availability

The data underlying this article will be shared upon reasonable request to the corresponding author.
