# Supplementary material for: Temporal dynamics of uterine immune microenvironment remodeling in a murine model of adenomyosis
Source: Mol Hum Reprod. 2025 Nov 26;31(4):gaaf057. doi: 10.1093/molehr/gaaf057 (PMC12714385; doi:10.1093/molehr/gaaf057)
Supplement: gaaf057_Supplementary_Data [file gaaf057_supplementary_data.pdf]

Temporal dynamics of uterine immune microenvironment remodeling in a murine model of adenomyosis.

Marlyne Squatrito, Julie Vervier, Laëtitia Bernet, Alessandra Camboni, Marie-Madeleine Dolmans, Carine Munaut.

Supplementary Figure S1. Flow cytometry gating strategy

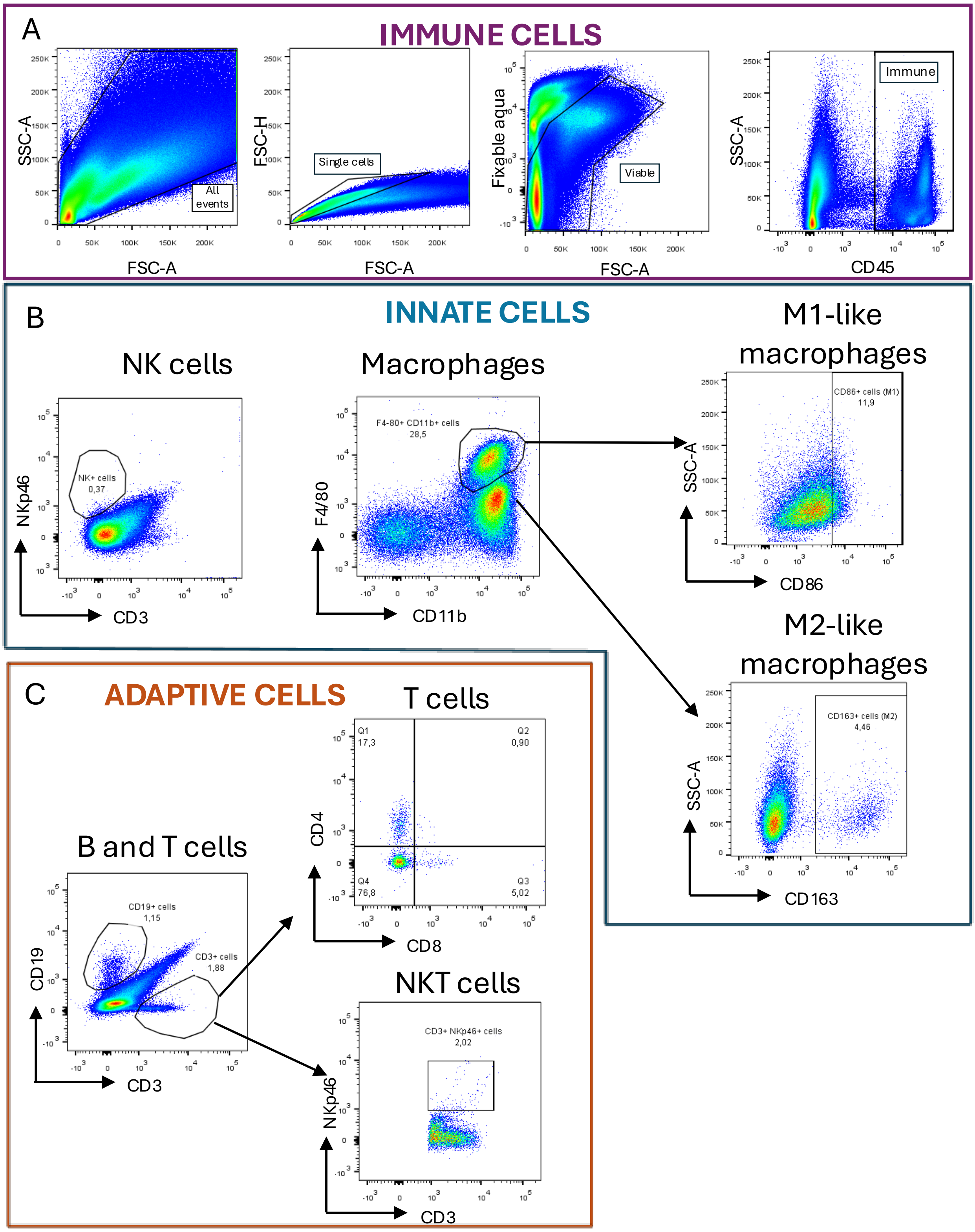

Supplementary Figure S1. Flow cytometry gating strategy. (A) Initial gating FSC/SSC, doublet exclusion (FSC-H/FSC-A), viability dye, and CD45+ immune cells. (B) Innate subsets: NK (CD3-NKp46+); macrophages (F4/80+CD11b+), further subdivided into M1-like (CD86+) and M2-like (CD163+). (C) Adaptive subsets: B cells (CD19+CD3-), T cells (CD19-CD3+), and T cell subpopulations (CD4/CD8: DN, $CD4^+$, $CD8^+$, DP). NKT cells defined as $CD3^+NKp46^+$. FMO controls were used to define gating thresholds but are not displayed here.
